# Inheritance of Nitrogen Use Efficiency in Inbred Progenies of Tropical Maize Based on Multivariate Diallel Analysis

**DOI:** 10.1155/2014/894710

**Published:** 2014-12-21

**Authors:** Fernando Lisboa Guedes, Rafael Parreira Diniz, Marcio Balestre, Camila Bastos Ribeiro, Renato Barbosa Camargos, João Cândido Souza

**Affiliations:** ^1^Embrapa Caprinos e Ovinos, 62030-497 Sobral, CE, Brazil; ^2^Departamento de Biologia, Universidade Federal de Lavras, 37200-000 Lavras, MG, Brazil; ^3^Departamento de Ciências Exatas, Universidade Federal de Lavras, 37200-000 Lavras, MG, Brazil; ^4^Departamento de Agricultura, Universidade Federal de Lavras, 37200-000 Lavras, MG, Brazil

## Abstract

The objective of our study was to characterize and determine the patterns of genetic control in relation to tolerance and efficiency of nitrogen use by means of a complete diallel cross involving contrasting inbred progenies of tropical maize based on a univariate approach within the perspective of a multivariate mixed model. Eleven progenies, previously classified regarding the tolerance and responsiveness to nitrogen, were crossed in a complete diallel cross. Fifty-five hybrids were obtained. The hybrids and the progenies were evaluated at two different nitrogen levels, in two locations. The grain yield was measured as well as its yield components. The heritability values between the higher and lower nitrogen input environment did not differ among themselves. It was observed that the general combining ability values were similar for both approaches univariate and multivariate, when it was analyzed within each location and nitrogen level. The estimate of variance of the specific combining ability was higher than general combining ability estimate and the ratio between them was 0.54. The univariate and multivariate approaches are equivalent in experiments with good precision and high heritability. The nonadditive genetic effects exhibit greater quantities than the additive genetic effects for the genetic control of nitrogen use efficiency.

## 1. Introduction

The maize production system in Brazil is quite varied in regard to the level of technology used. Modern production techniques with intensive application of inputs are used on many rural properties. However, there is a large group of typical family farm properties that produce 46% of Brazilian maize and in many cases they use little or no agricultural input [1]. This difference in the management system is clear in regard to fertilizer consumption, especially nitrogen fertilizers. In this context, new studies are of fundamental importance with a view toward generating more detailed information about the traits related to tolerance and responsiveness to nitrogen (N) use.

Investigation of the type of inheritance involved in these traits at different levels of N availability allows verification of which selection strategies would be most adequate for each environment. Studies of this nature are reported in the literature [2–4], but the results are not fully in agreement [5]. Consequently, validation of the type of inheritance requires more studies, as well as the use of more accurate statistical techniques.

Studies of the nature and magnitude of the genetic effects that control a given trait are based on phenotypic evaluations in multiple environments, followed by genetic/statistical analyses [3, 6]. These studies are important for the plant breeder because, that way, more adequate selection methods may be used to favorably exploit the types of genetic effects identified [2, 4, 7] in selection processes and prediction of hybrid behavior, as well as of segregating generations. For example, traits with predominantly additive inheritance could be evaluated in partially inbred progenies (third or fourth self-pollinated generation), or inbred lines evaluated “per se.” Traits in which the nonadditive effects are more important should preferentially be evaluated in crosses (top crosses, diallel crosses, interpopulational recurrent selection, etc.). Finally, traits with similar importance of additive and nonadditive effects could be evaluated by both strategies.

In obtaining superior genotypes, the evaluation of many traits allows inference of their relative superiority with greater precision. In application of biometric techniques, univariate analysis is normally used, with combined analyses generally restricted to bivariate procedures. Analysis of these variables in an isolated manner might not be sufficient to model the phenomenon for they do not consider the correlations existing between them. Thus, use of the theory of mixed model multivariate analysis allows combining the multiple pieces of information contained in the experimental unit so as to facilitate carrying out selection based on the combination of variables, allowing discrimination of the most promising genotypes [8].

In this context, the aim of this study was to characterize and determine the patterns of genetic control in relation to tolerance to and efficiency of nitrogen use by means of a complete diallel cross involving contrasting inbred progenies of tropical maize based on a univariate approach within the perspective of a multivariate mixed model.

## 2. Materials and Methods

The experiments were set up in the second week of November, in two locations during the 2011/2012 agricultural crop season. The experimental areas were designated as Envir 1 and Envir 2; the first one was located at 951 m altitude, 21°10′S and 44°55′W, while the second one was situated at 918 m altitude, 21°14′S and 45°00′W. In both areas no-tillage planting system was adopted and 22.15°C and 1,400 mm for average temperature and rainfall were registered, respectively [9].

Before sowing the experiments the main chemical attributes of these experimental areas were measured. The calculation of nitrogen amount applied on the experiments was done considering that 1% of organic matter in the soil equivalents to the liberation of 20 kg ha^−1^ of nitrogen, according to [10]. The other nutrients were corrected according to the necessity shown in the chemical analyses of the soil (Table 1).

At first, 67 progenies were obtained from the first noninbred generation after the selection was accomplished inside of a population in Hardy-Weinberg equilibrium (S_0:1_ progenies). These progenies were evaluated, originating from the germplasm bank of the Department of Biology of the Universidade Federal de Lavras (DBI/UFLA), Lavras, Minas Gerais, Brazil, in top cross combinations with two checks (one single hybrid and a mixture of the 67 progenies) at two levels of nitrogen. Among the progenies, based on the performance of the top crosses, six of the greatest tolerance and responsiveness to nitrogen (RT) and five of the least tolerance and responsiveness (R_n_T_n_) were chosen [11].

The 11 progenies selected were crossed in a complete diallel cross, synthesizing 55 hybrid combinations. Among them, 15 were derived from the crosses between RT progenies, 30 from the crosses between RT × R_n_T_n_ progenies, and 10 from the crosses between R_n_T_n_ progenies.

The 55 hybrids and the 11 S_0:2_ progenies, derived from self-pollination of the S_0:1_ progenies, were evaluated in experiments with different levels of nitrogen. For all the experiments, a randomized complete block design was used, with three replications. The plots consisted in two three-meter length rows, with a spacing of 0.6 m between rows and 0.25 m between plants, obtaining a density of approximately 66,666 plants ha^−1^.

For differentiation of the experiments in regard to nitrogen level, the following strategy was adopted: for experiments with high nitrogen availability (high N), fertilization was the same as recommended for the high technology level in Brazil [12, 13], which consisted in a total of 160 kg ha^−1^ of N. It was applied 28 kg ha^−1^ at planting, 42 kg ha^−1^ and 90 kg ha^−1^ in top dressing fertilization in the V4 and V8 phenological stage, respectively.

For the experiments with low nitrogen availability (low N), only fertilization at planting was carried out and consisted in what is previously described. All the other nutrients were added in the two experiments according to the crop recommendations.

The grain yield trait (t ha^−1^) was evaluated in all the experiments, as well as its yield components (ear length, ear diameter, cob diameter, and grain size), according to NiK et al. [14]. All the measurements of the yield components were obtained in centimeters.

First of all, analysis of variance was carried out for grain yield for the purpose of estimating selective accuracy and heritability. The estimate of selective accuracy (${\hat{r}}_{\hat{g}g}^{2}$) and the lower and upper limits were obtained according to de Resende [15].

Through the grain yield and yield component variables, two types of analysis of combining ability were made: one with a univariate focus, considering only the grain yield trait in an isolated manner, and the other with a multivariate focus, also considering the yield components. The SAS computational package [16] was used for that purpose.

The model adopted in this study, both for univariate and for multivariate analysis, respectively, was similar to that presented by Henderson and Quaas [17], however, with adaptation for analysis of combining ability given by

(1) $$\begin{array}{c} y_{i}=X_{i}\beta +Z_{1_{i}}\alpha +Z_{2_{i}}\omega +Z_{3_{i}}\phi +Z_{4_{i}}\tau +e_{i} \end{array}$$

in which *y*_*i*_ is the plot mean value in reference to trait *i* (*i* = 1, 2, 3, 4, and 5) evaluated in two locations and two levels of N, for a total of four environments; *X*_*i*_ is the incidence matrix of the fixed effects for the trait *n*; *Z*_1_*i*__ is the incidence matrix of the effects of the general combining ability for the trait *n*; *Z*_2_*i*__ is the incidence matrix of the effects of the specific combining ability for the trait *n*; *Z*_3_*i*__ is the incidence matrix of the interaction of the general combining abilities by environments for the trait *n*; *Z*_4_*i*__ is the incidence matrix of the interaction of the specific combining abilities by environments for the trait *n*. *β*, *α*, *ω*, *ϕ*, and *τ* are the vectors of the effects of *X*_*i*_, *Z*_1_*i*__, *Z*_2_*i*__, *Z*_3_*i*__, and *Z*_4_*i*__, respectively, and *e*_*i*_ is the random error of the model.

The multivariate model for combining ability is given by

(2) $$\begin{array}{c} y_{n}=X_{n}\beta +Z_{1_{n}}\alpha +Z_{2_{n}}\omega +Z_{3_{n}}\phi +Z_{4_{n}}\tau +e_{n} \end{array}$$

in which *y*_*n*_ is the plot mean value in reference to the *n*th trait (*n* = 1, 2, 3, 4, and 5) evaluated in two locations and two levels of N, for a total of four environments; *X*_*n*_ is the incidence matrix of the fixed effects for the trait *n*; *Z*_1_*n*__ is the incidence matrix of the effects of the general combining ability for the trait *n*; *Z*_2_*n*__ is the incidence matrix of the effects of the specific combining ability for the trait *n*; *Z*_3_*n*__ is the incidence matrix of the interaction of the general combining abilities by environments for the trait *n*; *Z*_4_*n*__ is the incidence matrix of the interaction of the specific combining abilities by environments for the trait *n*. *β*, *α*, *ω*, *ϕ*, and *τ* are the vectors of the effects of *X*_*n*_, *Z*_1_*n*__, *Z*_2_*n*__, *Z*_3_*n*__, and *Z*_4_*n*__, respectively, and *e*_*n*_ is the random error of the model. Expansion of the model was performed according to Balestre et al. [8].

For the purpose of better visualization of the behavior of the general combining abilities in relation to the environments and levels of nitrogen, graphs called GGE biplot (Genotype and Genotype by Environment Interaction) by Yan et al. [18] were plotted. The computational package SAS v 8.0 [16] was used to do so. For analysis of adaptability and stability, the *α*_1_ BLUPs were linearly combined with the *ϕ*_*ij*_ BLUPs so as to reconstruct the matrix *α*_1_ + *ϕ*_*ij*_. Thus, the simplified model of GCA + GCA_I_ is represented by

(3) $$\begin{array}{c} \alpha_{1}+\phi_{ij}=\lambda_{1}\gamma_{i1}\kappa_{j1}+\lambda_{2}\gamma_{i2}\kappa_{j2}+\rho_{ij} \end{array}$$

in which *α*_1_ is the BLUP mean of the general combining ability of parent *i* in environment *j*; *Ø*_*ij*_ is the BLUP of the interaction of general combining ability 1 in environment *j*; *λ*_1_ *γ*_*i*1_ *κ*_*j*1_ is the first principal component (PCA1) and picks up most of the effect *α*_1_ + *Ø*_*ij*_; *λ*_2_ *γ*_*i*2_ *κ*_*j*2_ is the second principal component (PCA2) and picks up the effect of genotypes (G) + interaction (GxA) of the complex type; *λ*_1_ and *λ*_2_ are the eigenvalues associated with the PCA1 and the PCA2; *γ*_*i*1_ and *γ*_*i*2_ are the scores of the PCA1 and the PCA2, respectively, for genotypes; *α*_*j*1_ and *α*_*j*2_ are the scores of the PCA1 and the PCA2, respectively, for environments; *ρ*_*ij*_ is the residue of the GCA × environment interaction, also known as “noise,” corresponding to the principal components not retained in the model.

The same procedure was adopted for SCA; that is,

(4) $$\begin{array}{c} \omega_{j}+\tau_{ij}=\lambda_{1}\gamma_{i1}\kappa_{j1}+\lambda_{2}\gamma_{i2}\kappa_{j2}+\rho_{ij}, \end{array}$$

in which *ω*_*j*_ is the BLUP mean of the specific combining ability *j*; *τ*_*ij*_ is the BLUP of the interaction of the specific combining ability *j*. The other components have the same definition as the previous model.

## 3. Results and Discussion

In the present study, selective accuracy statistics were obtained ranging from 82.22 to 91.92 (Table 2). Thus, the estimated parameters show high reliability. Through selective accuracy, inferences may also be made regarding the quality of an experiment. Values in this parameter above 0.70 and 0.90 are considered to be high and very high, respectively [19]; consequently, high experimental precision may be inferred.

It may be seen that the magnitudes of heritability for grain yield did not differ among themselves in the environments with high N or in those that received low N (Table 2). Soares et al. [20] found similar values for the coefficient of heritability at 0.78 and 0.83 for grain yield in environments with low and high availability of N, respectively.

The magnitudes of the GCA were similar in comparing the univariate and multivariate analyses within each environment and level of N (Table 2). This similarity in the estimates between the two approaches should probably be associated with good experimental precision, observed through selective accuracy and, consequently, through the high estimate of heritability (Table 2). Balestre et al. [8] observed that, under high heritability, there is no difference between the multivariate and univariate approach.

Nevertheless, it was observed that multivariate analysis exhibited lower mean quadratic error than univariate analysis, with magnitudes of 0.0663 and 0.2616, respectively (Table 2). This shows that greater precision in the GCA estimates may be obtained by multivariate analysis.

It may be observed that, of the six parents (G1 to G6) previously selected as RT [11], only G3 exhibited predictable behavior in regard to general combining ability in this study, with all estimates positive in high and low N (Table 2). As for the five parents selected as R_n_T_n_ (G7 to G11), three exhibited predictable behavior, with all the GCA estimates negative (G7, G8, and G10), and only two (G9 and G11) exhibited behavior different than expected, with all the GCA estimates positive and of greater magnitudes (Table 2). It is known that parents with positive GCA estimates contribute with a greater quantity of favorable alleles transmitted to the descendants [21]. In this context, G3 may be used in crosses with a view toward obtaining hybrids with greater tolerance and responsiveness to N. The parents 7, 8, and 10 have a lower frequency of favorable alleles, confirming their classification as R_n_T_n_. However, some parents exhibited behavior different than expected [11]. Thus, it may be inferred that early selection in S_0:1_ is not sufficient to fix favorable alleles to efficiency in N use. This shows that early selection for quantitative traits is only effective in cases in which the plants are extensively evaluated in various locations and with various replications [22].

It may be observed that there was no difference between the two analyses because there was a high genetic correlation of yield with the other traits, associated with high heritability. Therefore, little increase is expected through the use of multivariate analysis. Similar results were found by Balestre et al. [8] with common bean.

**Figure 1:**
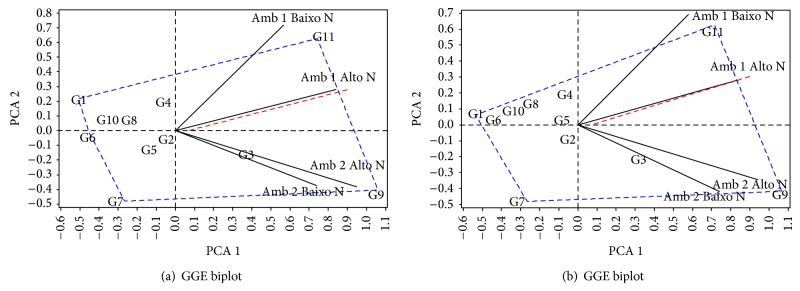
GGE biplot of univariate analysis (a) and multivariate analysis (b) with the first two principal components of GCA + GCA × Envir, corresponding to the representation of the eleven parents in two environments under high N and low N. In these figures, Amb, Alto, and Baixo refer to environment, high and low, respectively.

Differences were observed between the two environments (Envir 1 and Envir 2), which were separated into two megaenvironments by the red line traced in both the univariate and multivariate analyses (Figures 1(a) and 1(b)). This shows that the environments discriminate the genotypes evaluated in a different way.

In analysis of the first principal component (PCA1), it may be seen that the two environments, just as the two levels of N, showed positive scores in the two analyses (Figures 1(a) and 1(b)). It is clear from the figures that there was a greater performance difference in the GCA between the environments than between the levels; that is, the interaction is due to the difference of the environments. It may also be observed by the PCA1 that the high level N, in the two environments, showed greater magnitudes of scores. Thus, it may be affirmed that this level is better able to differentiate the parents than the low level N. DoVale et al. [2] affirm that, for both the hybrids and the parents in low N environments, there is less power of discrimination in these locations in regard to the effect of SCA and GCA.

Differentiation in the levels of N applied may also be observed. High level N, in the two environments, exhibited a lower score of the second principal component (PCA2) in relation to low level N in the two analyses (Figures 1(a) and 1(b)). In addition, on average, the general combining abilities were of greater magnitudes in high than in low level N; that is, the parents express a greater quantity of favorable alleles at high N. However, it is recommended that the parents be evaluated at both high and low N since the high and low N environments are related to two different characteristics, responsiveness and tolerance, respectively. Maia et al. [3] emphasize that these characteristics are controlled by different gene groups.

The results provided by analysis of the PCA1 and the PCA2 show the importance of always undertaking evaluation in contrasting environments because they express genetic variability in a different way [5]. Favorable environments allow better discrimination of the genotypes just as selection for responsiveness to N. Unfavorable environments, for their part, allow identification of genotypes tolerant to nitrogen deficiency.

It should be highlighted that the hybrids currently available on the market are developed under ideal soil fertility conditions, with a focus on high grain yield [5]. However, selection only in environments with high nitrogen availability may result in reduction of allele diversity associated with tolerance to low N availability [23]. A good cultivar to make available for planting would be one that aggregates both responsiveness and tolerance to nitrogen fertilization. That way, the cultivar would meet growing standards for properties of high and low technological level in regard to nitrogen fertilization.

In interpretation for parent performance, the PCA1 is related to adaptability; that is, it indicates those that have high GCA. In this respect, in the first megaenvironment, G11 was the most highly adapted in the two analyses (Figures 1(a) and 1(b)). Nevertheless, taking mean adaptability into consideration, it may be concluded that G9 was the one with closest to ideal performance since it was the one most adapted in the two megaenvironments.

The PCA2 indicates stability; that is, it is directly related to the interaction of the general combining abilities with environments. That way, parents with the PCA2 nearest to zero would be most stable in regard to GCA [18]. Therefore, in decreasing order, parents 6, 5, and 2 showed the best stabilities of GCA, but with negative signs (Figures 1(a) and 1(b)). Parents that showed high adaptability and accompanying stability were not observed; this fact indicates little participation of additive effects in efficiency of nitrogen use, as described above.

The importance of evaluating parents in contrasting environments in terms of N availability was shown, as well as the true importance of obtaining hybrids responsive to N and tolerant to the lack of N. Thus, in a breeding program for nitrogen use efficiency, the choice of parents for formation of base populations should take the adaptability and stability of the diallel analysis parameters into consideration. That way, one would be able to more precisely select parents that would add responsiveness and tolerance, regardless of N availability.

It may be observed that the estimate of variance of the SCA was greater than that of the GCA, and the ratio (GCA/SCA) was 0.54 (Table 3). Thus, it may be inferred that the nonadditive genetic effects exhibited greater quantities than the additive genetic effects. Similar results were reported by Gama et al. [24]. Nonadditive genetic effects in relation to dominance and/or epistasis were more determinant than the additive genetic effects in diverting the trend of general adaptability and leading to less predictable behaviors in the hybrid progenies [25]. For the grain yield characteristic, nonadditive genetic effects were predominant for environments with high and low N availability; however, when only the environment with high N availability was analyzed, it was seen that the additive effects were of greater importance [24].

Different results were found by Medici et al. [26], who showed that, in environments with high N availability, the additive genetic effects proved to be slightly more important than the nonadditive genetic effects. As for environments with low N availability, the additive and nonadditive genetic effects exhibited similar quantities. However, contradictory results are common in the literature and this fact is due to the differences in the germoplasm used, among other things [5].

The GCA × Envir interaction was of high magnitude (Table 3). This indicates that the quantity of favorable alleles donated by the parents changes among the environments. This corroborates results found by DoVale et al. [2] and DoVale et al. [27]. Thus, parents that promote an increase in the mean values of their hybrids evaluated in a certain environment and level of N might not contribute in a similar way when the hybrids are evaluated in another environment and level of N. This implies that there is low precision in the prediction of the behavior of the hybrids under different environments.

Nevertheless, the magnitude of the SCA × Envir interaction was low (Table 3), showing that, on average, the genetic complementation did not exhibit differences when related to hybrid performance in different environments and levels of N. Therefore, it was not possible to undertake biplot analysis. These results corroborate those found in the literature that report that the greater the heterozygosity of the genotypes is, the greater the phenotypic plasticity will be and, consequently, the greater the homeostasis will be [28, 29]. In this case, for the characteristics related to nitrogen use, selection based on the performance of the hybrid combination rather than on performance of the line per se is recommended. High genetic correlations coefficients among testcrosses at early and later inbreed generations were reported by Bernardo [30]. These coefficients increase as the difference between their inbreeding coefficients decreases, and it suggests that no difference is expected between the inbred and partially inbred progenies; therefore the general combining ability could be established using early generations. Through our results, while evaluating nitrogen use efficiency, the favorable alleles to grain yield were not fixed in the early selfing generations. This way, we may not state that a high genetic correlation would be observed between progenies S_0:1_ and their directly descended homozygous lines.

The mean variation of the SCAs exhibited small differences between the two univariate and multivariate analyses (Table 3). However, the hybrids originating from the RT × R_n_T_n_ group of progenies showed greater variation and magnitude in this parameter. This result shows that the best strategy for development of hybrids efficient in N use is to include at least one genotype responsive to N and also tolerant to deficiency of this nutrient in the cross. Similar results were reported by Tsai et al. [31] and Balko and Russel [32] when the authors evaluated inbred lines.

## 4. Conclusions

In experiments with good precision and high heritability, the univariate approach is equivalent to the multivariate approach. It indicates that the use of a statistic approach less complex, as univariate approach, should be chosen to facilitate the analysis of the dataset.

The best specific combining abilities arise from parents in contrast in regard to nitrogen use. This information is important to orientate breeders to choose genitors in order to obtain the best hybrids.

The general combining ability in noninbred progenies for efficiency in nitrogen use is influenced by the environment. It might be due to the difference in the expression of alleles under high and low N input, and in consequence the selection aim to obtain the best inbred lines should be done in specific environments.

The nonadditive genetic effects exhibit greater quantities than the additive genetic effects as it was observed in the SCA effects, in which it had a greater influence on tolerance and efficiency in nitrogen use. Thus, selection of the hybrid combination must be taken into consideration in selection of tolerant lines.

## Figures and Tables

**Table 1:** Chemical attributes of the soils collected where experiments with high level of nitrogen availability (high N) and low level of nitrogen availability (low N) were posteriorly set up.

Attributes	Experiments			
	High N		Low N	
	Envir 1	Envir 2	Envir 1	Envir 2
pH H_2_O	5.93	5.1	5.86	5.03
P (Melich) mg kg^−1^	5.53	9.63	8.1	36.73
K^+^ (cmolc kg^−1^)	57.66	90	56.66	116.00
Ca^2+^ (cmolc kg^−1^)	2.3	0.7	2.06	0.80
Mg^2+^ (cmolc kg^−1^)	0.9	0.2	0.83	0.30
Al^3+^ (cmolc kg^−1^)	0.1	0.33	0.1	0.30
H + Al (cmolc kg^−1^)	2.4	4.33	2.5	4.83
CEC-t (cmolc kg^−1^)	3.43	1.46	3.13	1.70
Organic matter (deg kg^−1^)	2.66	2.96	2.63	3.13

**Table 2:** Empirical BLUP estimation of the general combining abilities (GCA) in the univariate and multivariate approach, estimates of selective accuracy (${\hat{r}}_{\hat{g}g}^{2}$), heritability in the broad sense (${\hat{h}}_{a}^{2}$), and mean square error of the univariate and multivariate analyses of the grain yield trait (t ha^−1^) of the hybrids evaluated at different levels of N in two environments.

GCA								
Parents	Envir 1, high N		Envir 1, low N		Envir 2, high N		Envir 2, low N	
	Univariate	Multivariate	Univariate	Multivariate	Univariate	Multivariate	Univariate	Multivariate
G1	−0.3364	−0.3263	−0.1431	−0.3132	−0.7068	−0.6931	−0.3082	−0.2908
G2	−0.1758	−0.1800	0.0295	0.0169	0.1235	0.1233	−0.1232	−0.1220
G3	0.0548	0.0546	0.1671	0.1325	0.1703	0.1655	0.4317	0.4225
G4	0.0905	0.0887	0.0335	0.0124	−0.3173	−0.3206	0.0795	0.0812
G5	−0.0117	−0.0159	−0.1523	−0.0046	−0.2911	−0.2799	0.1608	0.1633
G6	−0.5829	−0.5800	−0.1520	−0.1290	−0.1693	−0.1665	−0.5136	−0.5117
G7	−0.2789	−0.2708	−0.5553	−0.5621	−0.0238	−0.0201	−0.1182	−0.1146
G8	−0.0497	−0.0438	−0.2478	−0.2481	−0.1792	−0.1761	−0.4435	−0.4541
G9	0.8006	0.8037	0.2987	0.3049	11.702	11.668	0.9185	0.9304
G10	−0.3165	−0.3145	−0.1357	−0.1306	−0.3443	−0.3441	−0.2740	−0.2654
G11	0.8061	0.7843	0.8574	0.8247	0.5677	0.5449	0.1902	0.1614

${\hat{r}}_{\hat{g}g}^{2}$	91.92		87.34		91.62		82.22	
${\hat{h}}_{a}^{2}$ (LL–UL)^¥^	84.45 (76.4–89.5)		76.29 (63.93–83.97)		83.93 (75.56–89.14)		67.66 (50.72–78.10)	
Mean square error								
	Univariate				Multivariate			

	0.2616				0.0663			

^*¥*^Lower limit (LL) and upper limit (UL) of the confidence interval of heritability, obtained at a level of 5% significance.

**Table 3:** Empirical BLUP estimation of the mean variation of the maximum and minimum values of the specific combining ability (SCA) of the hybrids synthesized between different groups of progenies, of the variances of the GCA and SCA and their interactions (GCA × Envir, SCA × Envir).

Mean variation of the SCA						
	RT^£^		RT × R_n_T_n_		R_n_T_n_	
	Univariate	Multivariate	Univariate	Multivariate	Univariate	Multivariate
Joint analyses	[−0.629; 0.609]^*^	[−0.644; 0.622]	[−0.643; 0.865]	[−0.647; 0.887]	[−0.360; 0.536]	[−0.323; 0.528]

Variances						
α_1_		ω_*j*_		*Ø*_*ij*_		τ_*ij*_

0,1702		0.3171		0.1080		0.0072

^£^(RT) hybrids synthesized between the responsive and tolerant progenies, (RT × R_n_T_n_) hybrids between responsive and tolerant progenies with nonresponsive and nontolerant progenies, and (R_n_T_n_) hybrids between nonresponsive and nontolerant progenies.

^*^Amplitudes of the empirical BLUP estimations.
